# Case Report: Adult NTRK-Rearranged Spindle Cell Neoplasm: Early Tumor Shrinkage in a Case With Bone and Visceral Metastases Treated With Targeted Therapy

**DOI:** 10.3389/fonc.2021.740676

**Published:** 2022-01-07

**Authors:** Federica Recine, Alessandro De Vita, Valentina Fausti, Federica Pieri, Alberto Bongiovanni, Eugenia Franchini, Roberto Casadei, Maria Cristina Falasconi, Devil Oboldi, Federica Matteucci, Maria Caterina Pallotti, Laura Mercatali, Nada Riva, Lorena Gurrieri, Silvia Vanni, Chiara Liverani, Giacomo Miserocchi, Chiara Spadazzi, Claudia Cocchi, Toni Ibrahim

**Affiliations:** ^1^ Osteoncology and Rare Tumors Center, IRCCS Istituto Romagnolo per lo Studio dei Tumori (IRST) “Dino Amadori”, Meldola, Italy; ^2^ Pathology Unit, Morgagni-Pierantoni Hospital, Forlì, Italy; ^3^ Biosciences Laboratory, IRCCS Istituto Romagnolo per lo Studio dei Tumori (IRST) “Dino Amadori”, Meldola, Italy; ^4^ Orthopedic Unit, Morgagni-Pierantoni Hospital, Forlì, Italy; ^5^ Rehabilitation Medicine Unit, Morgagni-Pierantoni Hospital, Forlì, Italy; ^6^ Radiology Unit, IRCCS Istituto Romagnolo per lo Studio dei Tumori (IRST) “Dino Amadori”, Meldola, Italy; ^7^ Nuclear Medicine Unit, IRCCS Istituto Romagnolo per lo Studio dei Tumori (IRST) “Dino Amadori”, Meldola, Italy; ^8^ Palliative Care Unit, IRCCS Istituto Romagnolo per lo Studio dei Tumori (IRST) “Dino Amadori”, Meldola, Italy

**Keywords:** NTRK-rearranged spindle cell neoplasm, NTRK fusions, oncogenic driver alterations, targeted therapies, visceral and bone involvement

## Abstract

**Background:**

NTRK (neurotrophic tyrosine receptor kinase)-rearranged spindle cell neoplasms are a new group of tumors included in the new 5^th^ edition of the World Health Organization (WHO) classification of soft Tissue and Bone Sarcomas. These tumors are characterized by NTRK gene fusions and show a wide spectrum of histologies and clinical behavior. Several targeted therapies have recently been approved for tumors harboring NTRK fusions, including STS.

**Case Presentation:**

A 26-year-old male with advanced, pretreated NTRK rearranged spindle cell neoplasm and liver, lung and bone metastases was treated with larotrectinib on a continuous 28-day schedule, at a dose of 100 mg twice daily. An ^18^FDG-PET/CT scan performed after 7 days of treatment showed tumor shrinkage in both visceral and bone lesions. There was no drug-related toxicity. Subsequent evaluations confirmed continued tumor regression in disease sites. The patient is well and continues treatment.

**Conclusion:**

The clinical and radiological response of our patient with an uncommon TPM4 (exon 7)-NTRK1 (exon 12) gene fusion tumor treated with a first-generation TRK inhibitor could contribute to a better understanding of the biology of this new STS entity and help to improve patient management.

## Introduction

Gene fusions corresponding to chromosomal rearrangements are believed to be involved in the pathogenetic mechanisms associated with various cancer types, including soft tissue sarcoma (STS) and bone sarcomas. Among sarcoma histotypes characterized by specific gene fusions, new clinical pathologic entities have emerged with specific histological and immunophenotypic features (1). NTRK (neurotrophic tyrosine receptor kinase)-rearranged spindle cell neoplasms represent novel rare STS recently included in the 5^th^ edition of the World Health Organization (WHO) Classification of Soft Tissue and Bone Sarcomas. These neoplasms, characterized by morphological and molecular features resembling lipofibromatosis-like neural tumors and peripheral nerve sheath tumors (PNSTs), show wide-ranging histologic grade, age at diagnosis, anatomic location and clinical behavior (2). The presence of NTRK fusion genes has been identified as the main oncogenic driver alteration leading to tumor pathogenesis in this new STS group.

Tropomyosin-related kinase (TRK) is a receptor tyrosine kinase family of neurotrophin receptors (NTs) expressed in human neuronal tissue (3). The TRK protooncogene family includes three proteins (TRKA, TRKB, and TRKC receptors) encoded by NTRK genes, including NTRK1, NTRK2 and NTRK3 genes, respectively. The correct regulation and activation TRK receptors are essential for normal cell function, and alterations of the TRK pathway may be involved in the pathogenesis of many cancer types. NTRK gene fusions are oncogenic drivers occurring across various adult and pediatric tumor histologies and have emerged as targets for cancer therapy (4–7).

A higher frequency of NTRK gene fusions has been reported in mammary analog secretory carcinoma, secretory breast carcinoma, infantile fibrosarcoma and congenital mesoblastic nephroma, occurring in > 90% of selected series (7–12). In other solid tumors, NTRK fusions are found at much lower frequencies, in particular about 0.2% in head and neck cancer, 0.2%-3.3% in lung cancer, 0.7%-1.5% in colorectal cancer, 0.3% in cutaneous melanoma 0.3% and 1% in sarcoma (13–15). Novel compounds have been developed to inhibit molecular alterations involving the TRK pathway, including larotrectinib (16–21). We report our “real life” experience of the clinical management of a young adult with metastatic pretreated NTRK-rearranged spindle cell neoplasm and high tumor burden who obtained a rapid response with larotrectinib, without side-effects and maintaining a good quality of life.

## Case Description

In 2007, a 14-year-old male underwent excision of an abdominal cutaneous lesion and was diagnosed with abdominal dermatofibrosarcoma protuberans (2 cm) with infiltrated surgical margins. The family history of the patient was unremarkable. The disease was limited to the abdomen. Radical resection was performed, with no evidence of residual disease. Further information about the surgery is lacking because the patient underwent diagnosis and treatment in another center. Eight years later, in January 2015, an ultrasound of the chest wall revealed a subcutaneous nodule of almost 5 cm. The lesion was radically excised, and histology revealed a metastasis compatible with dermatofibrosarcoma protuberans. Radiotherapy of the thorax was performed (60 Gy in 30 fractions) and follow-up was negative for one year. In 2016, a CT scan detected the presence of multiple bilateral nodules in the lung and two nodules in the liver, all suspicious for metastases. An ^18^FDG-PET/CT (18-fluorodeoxy-glucose positron emission tomography-computed tomography) scan showed areas of increased uptake in the 7^th^ and 8^th^ hepatic lobes and in 3 lung nodules. The patient began imatinib 400 mg/bid, decreased to 600 mg/die because of myelotoxicity. Disease progression was confirmed after 3 cycles and second-line treatment with anthracycline-based chemotherapy was proposed, which the patient refused. In 2017 the patient stopped all antitumor treatments and has since refused further clinical and imaging evaluations.

**Figure 1:**
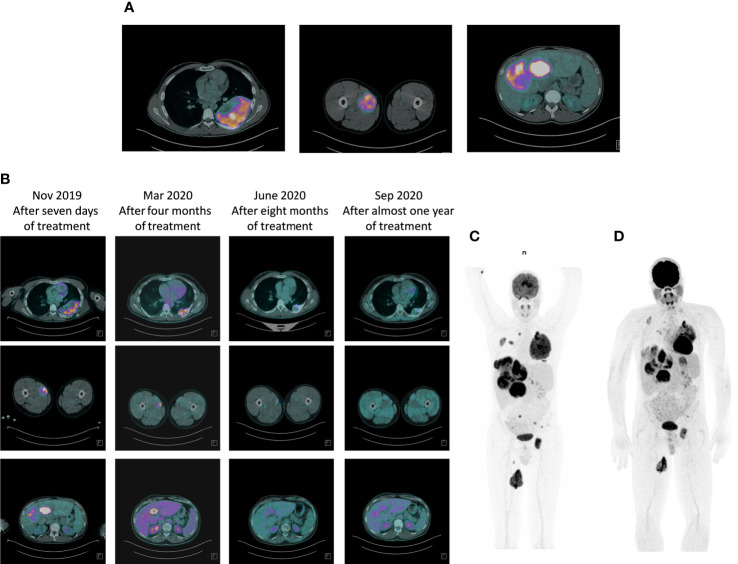
Response to therapy with larotrectinib: ^18^FDG-PET/CT scan evaluation. **(A)** Baseline FDG PET/CT study showing coronal fused PET/CT image with abnormal FDG uptake in multiple liver metastases, left rib and left femor. **(B)** Progressive metabolic response to larotrectinib after 7 days, four months, eight months and after almost one year of treatment. **(C)** Maximum intensity projection (MIP) PET/CT images: pre-therapy PET/CT scan again showed FDG-avid metastases occupying virtually the entire liver, left rib and left femor, all demonstrating high FDG avidity. **(D)** Seven days after the start of larotrectinib, PET/CT scan showed early tumor shrinkage with a reduction in FDG avidity in all previously described lesions.

In August 2019, the patient at the age of 26, was referred to our cancer institute from the Emergency Room of a local hospital following a diagnosis of bone metastases in the left acetabulum, with pathological fracture. A ^18^FDG-PET/CT scan revealed multiple bone metastases in the hip bone, bilateral ribs and left femur, multiple bilateral lung lesions, multiple liver metastases, muscle metastases, and one lesion in the left kidney suspicious for metastasis (**Figure 1A**). Radiotherapy was performed for the bone pathological fracture (20 Gy).

**Figure 2:**
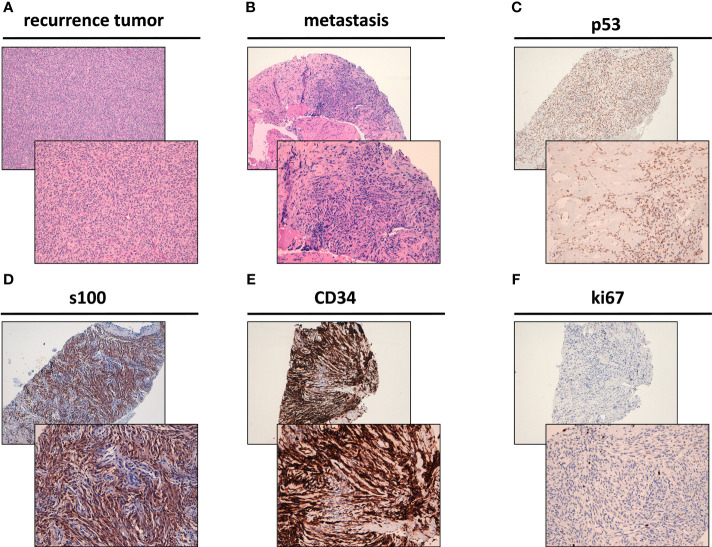
Histological features of NTRK rearranged spindle cell neoplasm. **(A)** Hematoxylin & Eosin (H&E) staining of the surgically resected recurrence tumor of the thoracic wall. (**B)** H&E staining of the surgically resected liver metastasis. **(C)** p53 positivity by immunohistochemistry **(**IHC) in the surgically resected liver metastasis. **(D)** S100 positivity by IHC in the surgically resected liver metastasis. **(E)** CD34 positivity by IHC in the surgically resected liver metastasis. **(F)** Ki-67 by IHC in the surgically resected liver metastasis (1% positivity).

The recurrence in the thoracic wall was composed of spindle cells characterized by low-grade morphology showing a mitotic rate of <5/10 high-power fields (HPFs) and low-to-moderate cellularity with focal vascular invasion (**Figure 2A**). Following a multidisciplinary evaluation of the case, a biopsy of the hepatic metastasis was performed, revealing the presence of spindle cells arranged in a clearly fascicular pattern (**Figure 2B**). The sections of the tumor were incubated with antibodies (22–24) and analyzed, revealing the expression of CD34, S100 and p53 at immunohistochemistry (IHC), Ki-67 1% (**Figures 2C** **–F**). These features were initially considered as confirmation of metastases form dermatofibrosarcoma protuberans.

**Figure 3:**
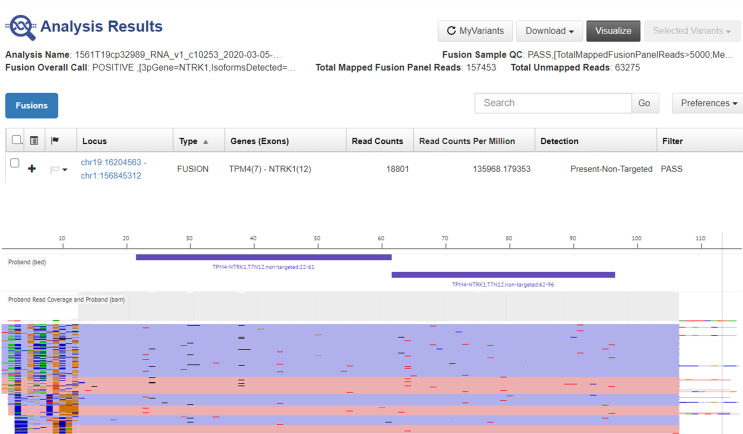
Representative graphical output of next-generation sequencing analysis using Oncomine Focus Assay showing TPM4-NTRK1 rearrangement in the liver metastasis.

**Figure 4:**
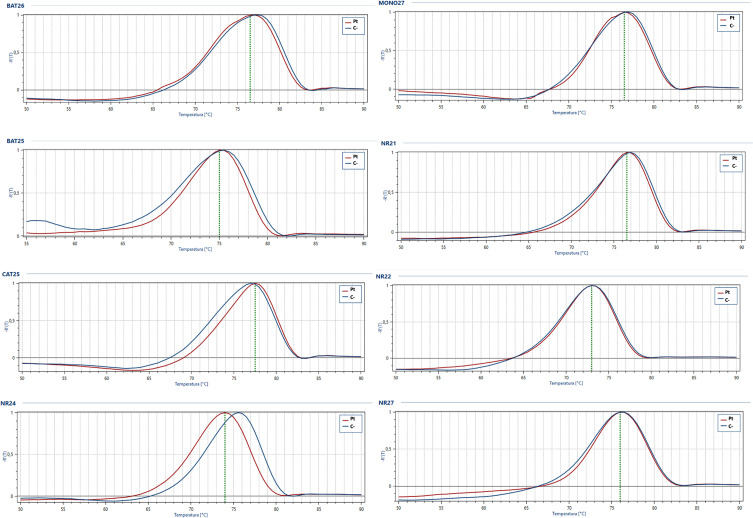
Microsatellite instability analysis. Representative melt curves of target gene analyzed for microsatellite instability status in patient tumor sample (Pt, red curve) compared to stable positive control (C-, blue curve).

Next generation sequencing (NGS) analysis was performed with the Oncomine Focus Assay on the histologic slides of the 2015 surgery to remove the subcutaneous nodule, highlighting the presence of the TPM4 (exon 7)-NTRK1 (exon 12) fusion gene (**Figure 3**). Microsatellite instability status (MSI) was also evaluated, but microsatellite stability was confirmed (**Figure 4**). Given the molecular findings, natural history of the disease and resistance to previous treatment, and after re-evaluation of the histological and immunohistochemical features of the case with an expert pathologist, it was concluded that the final diagnosis was compatible with the new entity of STS, NTRK-rearranged spindle cell neoplasm.

In November 2019, the patient started treatment with larotrectinib provided by Bayer as part of an early access program. Larotrectinib was administered orally in capsules on a continuous 28-day schedule, at a dose of 100 mg twice daily (**Figure 1B**). An ^18^FDG-PET/CT scan performed 7 days after the start of larotrectinib, considered as a baseline evaluation, showed early tumor shrinkage in both visceral and bone disease. The patient did not experience drug-related toxicity. Subsequent evaluations confirmed tumor regression in disease sites (**Figures 1C, D**). The last follow up of the patient was on October 2021 showing the confirmed metabolic response. The patient continues treatment, with no drug-related adverse events, and in good clinical conditions.

## Discussion

Soft tissue sarcomas (STS) are a heterogeneous group of mesenchymal tumors encompassing more than 80 histologic subtypes (1, 2). The clinical management of patients with STS should be decided by a multidisciplinary team and the histological diagnosis confirmed by an expert pathologist. The optimal therapeutic approach for advanced STS is still a much-debated issue, especially with regard to the best sequential treatments, mainly because of the high heterogeneity of these tumors (25) and the lack of clinical trials focusing on specific histotypes. The 5th edition of the WHO classification of Soft Tissue and Bone Sarcomas, published in early 2020, describes novel STS types, emphasizing the central role of morphology and selected genetic alterations in diagnostic and therapeutic approaches (2). NTRK-rearranged spindle cell neoplasms are included in this classification and represent an emerging group of STS characterized by a variety of morphologies and histological grades with wide-ranging clinical aggressiveness. These tumors occur mainly in the first two decades of life and are usually localized in the extremities or trunk. The recently described lipofibromatosis-like neural tumor (LPF-NT) and tumors resembling malignant peripheral nerve sheath tumors (MPNST) (11, 26, 27). From a histological point of view, NTRK-fusion cancers can be composed of spindle cells characterized by monomorphic spindle cell proliferation, an infiltrative pattern of growth, and frequent positivity for both S100 and CD34 proteins (28–30). Actually NTRK-fusion neoplasms show a high variety of morphologic patterns, including those similar to dermatofibrosarcoma protuberans-like.

In our case the first diagnosis was dermatofibrosarcoma protuberans but then the pathologist revised in NTRK-rearranged spindle cell neoplasm as the morphological, molecular features and clinical course of the disease.

NTRK1, NTRK2 and NTRK3 gene fusions are oncogenic drivers that represent a potential therapeutic target. NTRK fusions have been described in STS, which are morphologically distinct from infantile fibrosarcoma (IF), a locally aggressive mesenchymal tumor that rarely metastasizes and is typically characterized by ETV6-NTRK3 fusion. Several other NTRK fusion partners have also been identified (6, 7). NTRK1 rearrangement represents one of the most common genetic alterations in the group of NTRK-rearranged spindle cell neoplasms, showing more fusion partners than NTRK2 and NTRK3 genes (31). One of the most common NTRK fusions often involves LMNA gene, encoding nuclear envelope protein lamin A/C, and NTRK1, encoding neurotrophic receptor tyrosine kinase 1 in the MAP kinase signal pathway. The fusion results in a protein that activates the tyrosine kinase domain of the NTRK1 protein (1, 30). Other NTRK gene fusions involve tropomyosin 3 (TPM3) and translocated promoter region (TPR).

In our clinical case, we observed the presence of unusual NTRK fusion gene partners, *i.e.*, TPM4-NTRK1. To the best of our knowledge, this is a very rare chromosomal translocation in STS. Although NTRK3 fusion gene neoplasms are usually high grade and clinically aggressive, our patient’s tumor was characterized by low-grade morphology and a low mitotic rate over the course of several years, despite the appearance of metastatic disease (visceral and bone lesions). We can thus hypothesize a correlation with this uncommon NTRK gene fusion.

Several small-molecule targeted inhibitors of TRK tyrosine kinases are emerging on the therapeutic scenario, including larotrectinib. Data from case reports and phase 1 and 2 clinical studies have demonstrated the antitumor activity of this drug in TRK fusion-positive cancer, regardless of patient age and tumor type. Larotrectinib is an orally administrated, small-molecule inhibitor of all three TRK proteins. It was approved by the U.S. Food and Drug Administration and European Medicines Agency in 2018 for the so-called tumor-agnostic treatment of adult and pediatric patients with NTRK gene fusion solid tumors (16–21). The results from a recent pooled analysis of three phase 1/2 clinical trials enrolling adult and pediatric patients with metastatic TRK fusion-positive solid tumors confirmed that larotrectinib was highly active and safe in this setting (31).

We evaluated microsatellite instability (MSI) status to explore the potential presence of additional genetic mechanisms in this rare entity. MSI plays a role in some tumor types as a prognostic and predictive biomarker of response to immunotherapy (32–34) and results from an impaired DNA mismatch repair (MMR) system, whose job is to recognize and repair damage to DNA. The results from studies on the involvement of MSI in the STS tumorigenesis are somewhat contradictory, highlighting the need for further investigation (35–38). Our patient’s tumor showed microsatellite stability, suggesting that this specific molecular mechanism was not involved in his disease.

The disease evaluation at ^18^FDG-PET/CT showed an early tumor shrinkage that can be allocated in the category of partial response using RECIST criteria.

The rapid regression of the visceral and bone metastases seen in this uncommon case of TPM4-NTRK1 fusion-positive tumor provides important information on the clinical behavior of this new STS entity and contributes to establishing a new paradigm for the management of patients with NTRK-fusion cancers.

In conclusion, further preclinical research is warranted into this new entity because, in our opinion, primary cultures are the best strategy to understand the tumor biology of STS (22, 39–41).

## Data Availability Statement

The raw data supporting the conclusions of this article will be made available by the authors, without undue reservation.

## Ethics Statement

Ethical review and approval was not required for the study on human participants in accordance with the local legislation and institutional requirements. The patients/participants provided their written informed consent to participate in this study. Written informed consent was obtained from the individual(s) for the publication of any potentially identifiable images or data included in this article.

## Author Contributions

FR and TI conceived the idea for the paper. EF, LM, SV, CL, GM, CS, and CC performed the NGS and microsatellite analyses. ADV, VF, FP, and AB were responsible for data analysis. ADV, VF, AB, RC, NR, and LG interpreted the data. MCF, DO, FM, MCP, and LM performed image acquisitions. FR drafted the manuscript. All authors read and approved the final version of the manuscript for submission.

## Conflict of Interest

The authors declare that the research was conducted in the absence of any commercial or financial relationships that could be construed as a potential conflict of interest.

## Publisher’s Note

All claims expressed in this article are solely those of the authors and do not necessarily represent those of their affiliated organizations, or those of the publisher, the editors and the reviewers. Any product that may be evaluated in this article, or claim that may be made by its manufacturer, is not guaranteed or endorsed by the publisher.

## References

[1] Miettinen M Felisiak-Golabek A Luiña Contreras A Glod J Kaplan RN Lasota J. New Fusion Sarcomas: Histopathology and Clinical Significance of Selected Entities. Hum Pathol (2019) 86:57–65. doi: 10.1016/j.humpath.2018.12.006 30633925 PMC7443750

[2] Fletcher CDM Unni KK Mertens F. WHO Classification of Tumours Editorial Board. WHO Classification of Tumours of Soft Tissue and Bone, 5th Ed. Lyon, France: IARC Press (2020).

[3] Nakagawara A. Trk Receptor Tyrosine Kinases: A Bridge Between 27. Cancer and Neural Development. Cancer Lett (2001) 169(2):107–14. doi: 10.1016/s0304-3835(01)00530-4 11431098

[4] Huang EJ Reichardt LF. Trk Receptors: Roles in Neuronal Signal Transduction. Annu Rev Biochem (2003) 72:609–42. doi: 10.1146/annurev.biochem.72.121801.161629 12676795

[5] Boulle F Kenis G Cazorla M Hamon M Steinbusch HWM Lanfumey L. TrkB Inhibition as a Therapeutic 28. Target for CNS-Related Disorders. Prog Neurobiol (2012) 98:197–206. doi: 10.1016/j.pneurobio.2012.06.002 22705453

[6] Amatu A Sartore-Bianchi A Siena S. NTRK Gene Fusions as Novel Targets of Cancer Therapy Across Multiple Tumour Types. ESMO Open (2016) 1(2):e000023. doi: 10.1136/esmoopen-2015-000023 27843590 PMC5070277

[7] Drilon A. TRK Inhibitors in TRK Fusion-Positive Cancers. Ann Oncol (2019) 30(Suppl_8):viii23–30. doi: 10.1093/annonc/mdz282 PMC6859818 31738426

[8] Drilon A Wang L Hyman DM Hechtman J Wei G Cam NR. What Hides Behind the MASC: Clinical Response and Acquired Resistance to Entrectinib After ETV6-NTRK3 Identification in a Mammary Analogue Secretory Carcinoma (MASC). Ann Oncol (2016) 27(5):920–6. doi: 10.1093/annonc/mdw042 PMC4843186 26884591

[9] Halalsheh H McCarville MB Neel M Reynolds M Cox MC Pappo AS. Dramatic Bone Remodeling Following Larotrectinib Administration for Bone Metastasis in a Patient With TRK Fusion Congenital Mesoblastic Nephroma. Pediatr Blood&Cancer (2018) 65(10):e27271. doi: 10.1002/pbc.27271 29893456

[10] Tognon C Becker L Carneiro F MacPherson N Horsman D Poremba C. Expression of the ETV6-NTRK3 Gene Fusion as a Primary Event in Human Secretory Breast Carcinoma. Cancer Cell (2002) 2:367–76. doi: 10.1016/S1535-6108(02)00180-0 12450792

[11] Davis JL Lockwood CM Albert CM Tsuchiya K Hawkins DS Rudzinski ER. Infantile NTRK-Associated Mesenchymal Tumors. Pediatr Dev pathol: Off J Soc Pediatr Pathol Paediatric Pathol Soc (2018) 21:68–78. doi: 10.1177/1093526617712639 28683589

[12] Laetsch TW Nagasubramanian R Davis JL Rudzinski E Feraco AM Tuch BB. Larotrectinib for Paediatric Solid Tumours Harbouring NTRK Gene Fusions: Phase 1 Results From a Multicentre, Open-Label, Phase 1/2 Study. Lancet Oncol (2018) 19:705–14. doi: 10.1016/S1470-2045(18)30119-0 PMC5949072 29606586

[13] Vaishnavi A Le AT Doebele RC. TRKing Down an Old Oncogene in a New Era of Targeted Therapy. Cancer Discov (2015) 5:25–34. doi: 10.1158/2159-8290.CD-14-0765 25527197 PMC4293234

[14] Stransky N Cerami E Schalm S Kim JL Lengauer C. The Landscape of Kinase Fusions in Cancer. NatCommun (2014) 5:4846. doi: 10.1038/ncomms5846 PMC4175590 25204415

[15] Ardini E Bosotti R Borgia AL Amboldi N Raddrizzani L Milani A. The TPM3-NTRK1 Rearrangement is a Recurring Event in Colorectal Carcinoma and is Associated With Tumor Sensitivity to TrkA Kinase Inhibition. Mol Oncol (2014) 8:1495–507. doi: 10.1016/j.molonc.2014.06.001 PMC5528583 24962792

[16] Doebele RC Davis LE Vaishnavi A Le AT Estrada-Bernal A Keysar S. An Oncogenic NTRK Fusion in a Patient With Soft-Tissue Sarcoma With Response to the Tropomyosin-Relatedkinaseinhibitor LOXO-101. Cancer Discov (2015) 5:1049e1057. doi: 10.1158/2159-8290.CD-15-0443 26216294 PMC4635026

[17] Landman Y Ilouze M Wein S Neiman V Yerushalmi R Yakimov M. Rapid Response to Larotrectinib (LOXO-101) in an Adult Chemotherapy-Naive Patients With Advanced Triple-Negative Secretory Breast Cancer Expressing ETV6-NTRK3 Fusion. Clin Breast Cancer (2018) 18:e267ee270. doi: 10.1016/j.clbc.2017.11.017 29233640

[18] Nagasubramanian R Wei J Gordon P Rastatter JC Cox MC Pappo A. Infantile Fibrosarcoma With NTRK3-ETV6 Fusion Successfully Treated With the Tropomyosin-Related Kinase Inhibitor LOXO-101. Pediatr Blood&Cancer (2016) 63:1468e1470. doi: 10.1002/pbc.26026 PMC5074243 27093299

[19] Ziegler DS Wong M Mayoh C Kumar A Tsoli M Mould E. Brief Report: Potentclinical and Radiologicalresponse to Larotrectinib in TRK Fusiondriven High-Grade Glioma. Br J Cancer (2018) 119:693e696. doi: 10.1038/s41416-018-0251-2 30220707 PMC6173734

[20] Laetsch TW DuBois SG Mascarenhas L Nagasubramanian R Davis JL Rudzinski E. Larotrectinib for Paediatric Solid Tumoursharbouring NTRK Gene Fusions: Phase 1 Results From a Multicentre, Open-Label, Phase 1/2 Study. Lancet Oncol (2018) 19:705–14. doi: 10.1016/S1470-2045(18)30119-0 PMC5949072 29606586

[21] Drilon A Laetsch TW Kummar S Nathenson M Doebele RC Farago AF. Efficacy of Larotrectinib in TRK Fusion-Positive Cancers in Adults and Children. N Engl J Med (2018) 378:731–9. doi: 10.1056/NEJMoa1714448 PMC5857389 29466156

[22] De Vita A Recine F Miserocchi G Pieri F Spadazzi C Cocchi C. The Potential Role of the Extracellular Matrix in the Activity of Trabectedin in UPS and L-Sarcoma: Evidences From a Patient-Derived Primary Culture Case Series in Tridimensional and Zebrafish Models. J Exp Clin Cancer Res (2021) 40(1):165. doi: 10.1186/s13046-021-01963-1 33975637 PMC8111914

[23] De Vita A Recine F Mercatali L Casadei R Bongiovanni A Pieri F. Myxofibrosarcoma Primary Cultures: Molecular and Pharmacological Profile. Ther Adv Med Oncol (2017) 9:755–67. doi: 10.1177/1758834017737472 PMC5808841 29449896

[24] De Vita A Recine F Mercatali L Bongiovanni A Pieri F Casadei R. Primary Culture of Undifferentiated Pleomorphic Sarcoma: Molecular Characterization and Response to Anticancer Agents. Int J Mol Sci (2017) 18:2662. doi: 10.3390/ijms18122662 PMC5751264 29292724

[25] Martín-Broto J Reichardt P Jones RL Stacchiotti S. Different Approaches to Advanced Soft Tissue Sarcomas Depending on Treatment Line, Goal of Therapy and Histological Subtype. Expert RevAnticancerTher (2020) 20(sup1):15–28. doi: 10.1080/14737140.2020.1753510 32349558

[26] Racanelli D Brenca M Baldazzi D Goeman F Casini B De Angelis B. Next-Generation Sequencing Approaches for the Identification of Pathognomonic Fusion Transcripts in Sarcomas: The Experience of the Italian ACC Sarcoma Working Group. Front Oncol (2020) 10:489. doi: 10.3389/fonc.2020.00489 32351889 PMC7175964

[27] Agaram NP Zhang L Sung YS Fletcher CD. Recurrent NTRK1 Gene Fusions Define a Novel Subset of Locally Aggressive Lipofibromatosis- Like Neural Tumors. Am J SurgPathol (2016) 40:1407–16. doi: 10.1097/PAS.0000000000000675 PMC5023452 27259011

[28] Suurmeijer AJ Dickson BC Swanson D Zhang L Sung Y-S Huang H-Y. The Histologic Spectrum of Soft Tissue Spindle Cell Tumors With NTRK3 Gene Rearrangements. Genes Chromosomes Cancer (2019) 58(11):739–46. doi: 10.1002/gcc.22767 PMC6733642 31112350

[29] Antonescu CR Dickson BC Swanson D Zhang L Sung YS Kao YC. Spindle Cell Tumors With RET Gene Fusions Exhibit a Morphologic Spectrum Akin to Tumors With NTRK Gene Fusions. Am J Surg Pathol (2019) 43(10):1384–91. doi: 10.1097/PAS.0000000000001297 PMC6742579 31219820

[30] Haller F Knopf J Ackermann A Moskalev EA Will R Satir AA. Paediatric and Adult Soft Tissue Sarcomas With NTRK1 Gene Fusions: A Subset of Spindle Cell Sarcomas Unified by a Prominent Myopericytic/ Haemangiopericytic Pattern. J Pathol (2016) 23:700–10. doi: 10.1002/path.4701 26863915

[31] Hong DS Du Bois SG Kummar S Farago F Albert CM Rohrberg KS. Larotrectinib in Patients With TRK Fusion-Positive Solid Tumours: A Pooled Analysis of Three Phase 1/2 Clinical Trials. Lancet Oncol (2020) 21(4):531–40. doi: 10.1016/S1470-2045(19)30856-3 PMC7497841 32105622

[32] Boland CR Goel A. Microsatelliteinstability in Colorectalcancer. Gastroenterology (2010) 138:2073–87.e3. doi: 10.1053/j.gastro.2009.12.064 20420947 PMC3037515

[33] Miquel C Jacob S Grandjouan S Aimé A Viguier J Sabourin J-C. Frequent Alteration of DNA Damage Signalling and Repair Pathways in Human Colorectal Cancers With Microsatellite Instability. Oncogene (2007) 26:5919–26. doi: 10.1038/sj.onc.1210419 17384679

[34] Thomas J Leal A Overman MJ. Clinical Development of Immunotherapy for Deficient Mismatch Repair Colorectal Cancer. Clin Colorectal Cancer (2020) 19(2):73–81. doi: 10.1016/j.clcc.2020.02.002 32173280

[35] Rucinska M Kozłowski L Pepiński W Skawrońska M Janica J Wojtukiewic MZ. High-Grade Sarcomas are Associated With Microsatellite Instability (Chromosome 12) and Loss of Heterozygosity (Chromosome 2). Med Sci Monit (2005) 11:BR65–8. 15668629

[36] Kawaguchi K Oda Y Takahira T Saito T Yamamoto H Kobayashi C. Microsatellite Instability and Hmlh1 and Hmsh2 Expression Analysis in Soft Tissue Sarcomas. Oncol Rep (2005) 13:241–6. doi: 10.3892/or.13.2.241 15643505

[37] Monument MJ Lessnick SL Schiffman JD Randall RT. Microsatellite Instability in Sarcoma: Fact or Fiction? ISRN Oncol (2012) 2012:473146. doi: 10.5402/2012/473146 23401795 PMC3564276

[38] De Vita A Vanni S Fausti V Cocchi C Recine F Miserocchi G. Deciphering the Genomic Landscape and Pharmacological Profile of Uncommon Entities of Adult Rhabdomyosarcomas. Int J Mol Sci (2021) 22(21):11564. doi: 10.3390/ijms222111564 34768995 PMC8584142

[39] De Vita A Mercatali L Miserocchi G Bongiovanni A Pieri F Cavaliere D. Establishment of a Primary Culture of Patient-Derived Soft Tissue Sarcoma. J Vis Exp (2018) 11:56767. doi: 10.3791/56767 PMC5933451 29708525

[40] Miserocchi G Mercatali L Liverani C De Vita A Spadazzi C Pieri F. Management and Potentialities of Primary Cancer Cultures in Preclinical and Translational Studies. J Transl Med (2017) 15:229. doi: 10.1186/s12967-017-1328-z 29116016 PMC5688825

[41] De Vita A Miserocchi G Recine F Bongiovanni A Cavaliere D Liverani C. Activity of Eribulin in a Primary Culture of Well-Differentiated/Dedifferentiated Adipocytic Sarcoma. Molecules (2016) 21:1662. doi: 10.3390/molecules21121662 PMC6273088 27918490

